# Identification of aberrantly methylated differentially expressed genes in prostate carcinoma using integrated bioinformatics

**DOI:** 10.1186/s12935-019-0763-8

**Published:** 2019-03-05

**Authors:** Kai Wu, Xiaotao Yin, Yipeng Jin, Fangfang Liu, Jiangping Gao

**Affiliations:** 1 Department of Urology, Chinese PLA General Hospital, Beijing, China; 2 Department of Urology, First Affiliated Hospital of Chinese PLA General Hospital, Beijing, China; 3 Hebei General Hospital of Civil Affairs, Xingtai, Hebei Province China

**Keywords:** Prostate cancer, Methylation, Expression, Bioinformatics

## Abstract

**Background:**

Methylation plays a key role in the aetiology and pathogenesis of prostate cancer (PCa). This study aimed to identify aberrantly methylated differentially expressed genes (DEGs) and pathways in PCa and explore the underlying mechanisms of tumourigenesis.

**Methods:**

Expression profile (GSE29079) and methylation profile (GSE76938) datasets were obtained from the Gene Expression Omnibus (GEO). We used R 3.4.4 software to assess aberrantly methylated DEGs. The Cancer Genome Atlas (TCGA) RNA sequencing and Illumina HumanMethylation450 DNA methylation data were utilized to validate screened genes. Functional enrichment analysis of the screened genes was performed, and a protein–protein interaction (PPI) network was constructed using the Search Tool for the Retrieval of Interacting Gens (STRING). The results were visualized in Cytoscape. After confirmation using TCGA, cBioPortal was used to examine alterations in genes of interest. Then, protein localization in PCa cells was observed using immunohistochemistry.

**Results:**

Overall, 536 hypomethylated upregulated genes were identified that were enriched in biological processes such as negative regulation of transcription, osteoblast differentiation, intracellular signal transduction, and the Wnt signalling pathway. Pathway enrichment showed significant changes in factors involved in AMPK signalling, cancer, and adherens junction pathways. The hub oncogenes were *AKT1*, *PRDM10*, and *FASN*. Additionally, 322 hypermethylated downregulated genes were identified that demonstrated enrichment in biological processes including positive regulation of the MAPK cascade, muscle contraction, ageing, and signal transduction. Pathway analysis indicated enrichment in arrhythmogenic right ventricular cardiomyopathy (ARVC), focal adhesion, dilated cardiomyopathy, and PI3K-AKT signalling. The hub tumour suppressor gene was *FLNA*. Immunohistochemistry showed that *AKT1*, *FASN*, and *FLNA* were mainly expressed in PCa cell cytoplasm, while *PRDM10* was mainly expressed in nuclei.

**Conclusions:**

Our results identify numerous novel genetic and epigenetic regulatory networks and offer molecular evidence crucial to understanding the pathogenesis of PCa. Aberrantly methylated hub genes, including *AKT1*, *PRDM10*, *FASN,* and *FLNA*, can be used as biomarkers for accurate PCa diagnosis and treatment. In conclusion, our study suggests that *AKT1*, *PRDM10*, and *FASN* may be tumour promoters and that *FLNA* may be a tumour suppressor in PCa. We hope these findings will draw more attention to these hub genes in future cancer studies.

## Background

Prostate cancer (PCa) is the second most common malignant tumour in males, and it is the fifth leading cause of cancer mortality [1]. Due to prostate-specific antigen (PSA) screening and advanced biopsy techniques, early-stage PCa patients often show good prognosis after comprehensive treatment. However, PCa is a latent disease and can occur as an asymptomatic tumour in 20- to 30-year-old men [2]. The disease becomes symptomatic in the advanced stage, at which point fewer effective treatment options are available than at earlier stages [1]. Consequently, the overall survival (OS) of patients with advanced PCa is significantly diminished [3, 4]. Therefore, new specific biomarkers for early PCa detection are urgently needed.

In recent years, tumour epigenetic modifications, acknowledged as inherited modifications in gene expression, including DNA methylation, histone acetylation, and noncoding RNA-related modifications, have garnered significant research interest [5]. As the main epigenetic modification, DNA methylation has been extensively studied with respect to angiogenesis, apoptosis, cell cycle regulation, and DNA damage repair [6, 7]. Abnormal methylation, including hypomethylation of oncogenes and hypermethylation of tumour suppressor genes, is intimately involved in tumour pathogenesis and is significantly correlated with patient survival in many cancers [8, 9].

Genetic testing based on microarray and sequencing platforms has emerged as a promising and effective tool to screen significant genetic or epigenetic changes in carcinogenesis and to identify biomarkers useful in diagnosis and prognosis determination [10]. A number of differentially expressed genes (DEGs) and differentially methylated genes (DMGs) have been identified in PCa though microarray analysis [11, 12]. Although some studies have focused on specific genes with aberrant DNA hypermethylation or hypomethylation in PCa, an integrated analysis of gene expression, methylation, and signalling pathway interactions has not been performed.

In the present study, we assessed the interaction network of DEGs and DMGs along with interrelated signalling pathways in PCa by analysing gene expression microarray data (GSE29079), gene methylation microarray data (GSE76938), oncogenes, and tumour suppressor genes (TSGs) using bioinformatic tools; such an analysis has not been reported in previous research. We then used a dataset from the Cancer Genome Atlas (TCGA) as a validation cohort for our findings. We aimed to provide novel insights into the biological characteristics and pathways of DEGs/DMGs in PCa and to identify putative biomarkers important for the development and progression of PCa. Target genes such as AKT1, PRDM10, FASN, and FLNA may play essential roles in the diagnosis and treatment of PCa.

## Materials and methods

### Microarray data

In the current study, a gene expression dataset (GSE29079) and a gene methylation dataset (GSE76938) were downloaded from the Gene Expression Omnibus (GEO, https://www.ncbi.nlm.nih.gov/geo/). In total, 47 PCa and 48 normal prostate specimens were registered in GSE29079 (platform: GPL5175 Affymetrix Human Exon 1.0 ST Array). The gene methylation microarray dataset, GSE76938, was composed entirely of 63 normal prostate tissues and 73 PCa tumour tissues (GPL13534 Illumina HumanMethylation450 BeadChip).

### Data processing

The expression and methylation were analysed with R 3.4.4 software (https://www.r-project.org/). To analyse the DEGs, we used a P < 0.05 and a |t| > 2 as cut-off standards. For the DMGs, we selected an FDR < 0.05 and a β > 0.2 as cut-off values. We obtained an oncogene list from the ONGene database (http://ongene.bioinfo-minzhao.org/) and a tumour suppressor gene (TSG) list from the TSGene database (https://bioinfo.uth.edu/TSGene/index.html). Finally, hypomethylated highly expressed genes were identified based on the overlapping hypomethylated and upregulated genes. Similarly, hypermethylated genes with low expression were identified based on the overlapping hypermethylated and downregulated genes. We also used the VennDiagram package in R software to identify overlapping DEGs, DMGs, oncogenes, and TSGs.

### Functional and pathway enrichment analysis

The Database for Annotation, Visualization, and Integrated Discovery (DAVID, https://david.ncifcrf.gov/) was used to perform Gene Ontology (GO) enrichment analysis. DAVID is an online tool for systematic and integrative annotation and enrichment analysis that can be used to reveal biological meaning related to large gene lists [13]. GO analysis for the cellular component, biological process (BP), and molecular function (MF) categories [14] and Kyoto Encyclopedia of Genes and Genomes (KEGG) pathway enrichment analysis [15] were performed for the selected genes (hypomethylated genes with high expression and hypermethylated genes with low expression) using the DAVID. A p value < 0.01 was considered statistically significant.

### Protein–protein interaction (PPI) network generation and module analysis

The Search Tool for the Retrieval of Interacting Genes (STRING) is an online database used to predict PPIs, which are essential for interpreting the molecular mechanisms of key cellular activities in carcinogenesis. In this study, we used the STRING database to build a PPI network of hypomethylated genes with high expression and hypermethylated genes with low expression. The cut-off standard was defined as an interaction score of 0.4. The target hub genes used for further analysis had to meet the following 2 criteria: (i) they were oncogenes/TSGs; and (ii) they were in the top 30 genes according to 5 cytoHubba ranking methods using Cytoscape software. Subsequently, the PPI network was visualized by Cytoscape, and the Molecular Complex Detection (MCODE) algorithm in Cytoscape software was used to screen modules. An MCODE score > 3 and a node number > 5 were taken as the criteria to define a module.

### Validation of the target genes in TCGA

To confirm the results, a dataset was downloaded from TCGA to validate the methylation and expression levels of the genes of interest. TCGA includes comprehensive, multi-dimensional maps of key genomic changes in various types of cancers. Additionally, the translational levels of the hub genes were validated using the Human Protein Atlas (HPA) database. The cBio Cancer Genomics Portal, an open platform for analysing large-scale cancer genomics datasets for various cancers, was used to explore the genetic alterations connected to the hub genes and to examine the correlations between mRNA expression and DNA methylation in PCa.

### Immunohistochemistry

Prostate tissue arrays including normal prostate tissue samples and PCa samples were purchased from Cybrdi (Gaithersburg, MD). The PR482a array contains 48 dot samples from 24 cases. The pathological diagnoses of the array samples are described in the manufacturer’s product sheet (http://www.alenabio.com/public/details?productId=58422&searchText=).After deparaffinization in xylene and rehydration, the sections were incubated in 4% H_2_O_2_ for 10 min to block endogenous peroxidase activity. After blocking the samples with 15% goat serum in phosphate-buffered saline (PBS) for 30 min, the sections were separately incubated at 4 °C overnight with antibodies against *AKT1* (Proteintech Group, Inc.), *FASN* (Proteintech Group, Inc.), *PRDM10* (Biosynthesis Biotechnology, Inc.) and *FLNA* (Proteintech Group, Inc.). Images were captured on a Pannoramic MIDI microscope.

## Results

### Identification of aberrantly methylated DEGs in PCa

**Fig. 1:**
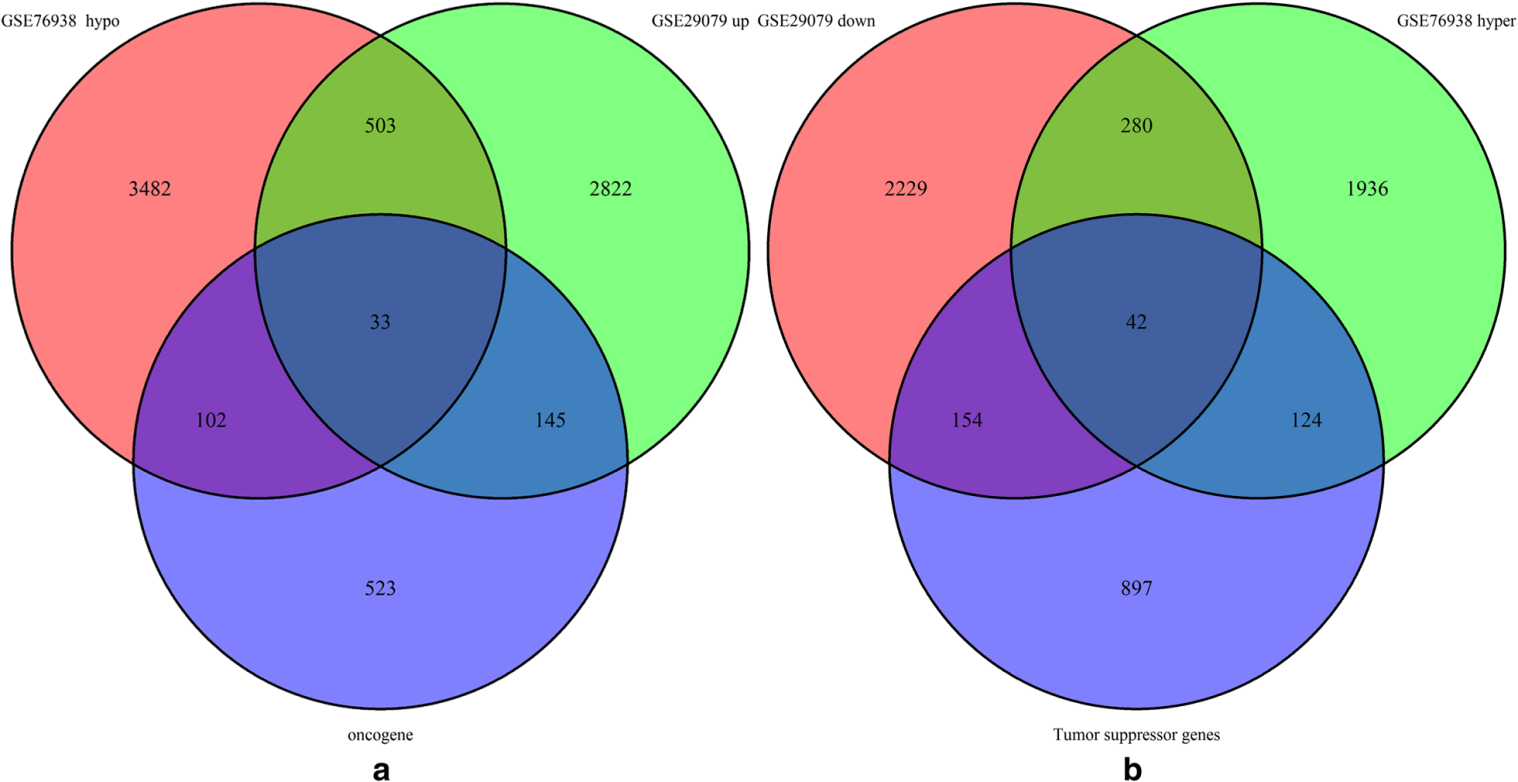
Identification of aberrantly methylated differentially expressed genes and related oncogenes and tumour suppressor genes (TSGs). **a** A total of 322 upregulated hypomethylated genes were identified, 42 of which were oncogenes. **b** A total of 533 downregulated hypermethylated genes were identified, 33 of which were TSGs

**Fig. 2:**
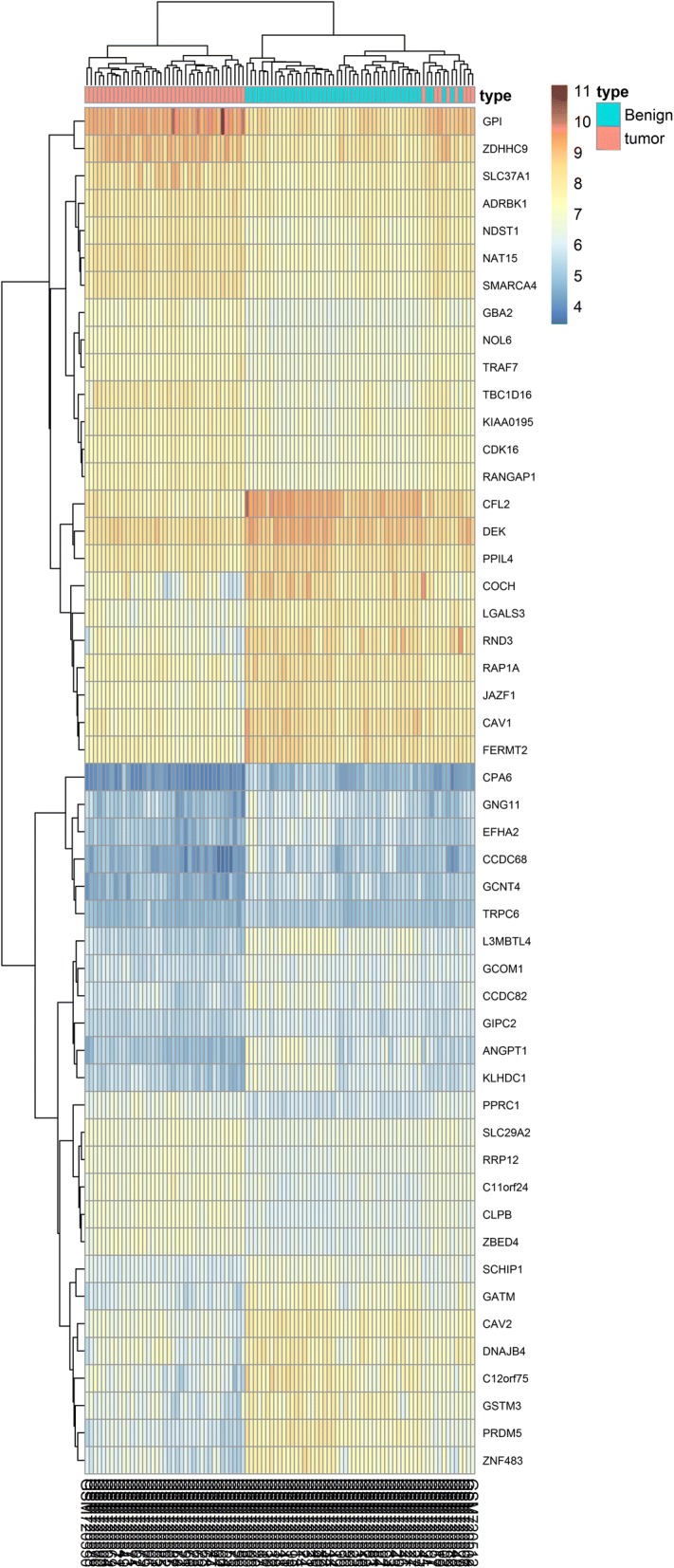
Clustered heat map of the top 50 differentially expressed genes (DEGs) in GSE29079. Red: upregulated genes; blue: downregulated genes

**Fig. 3:**
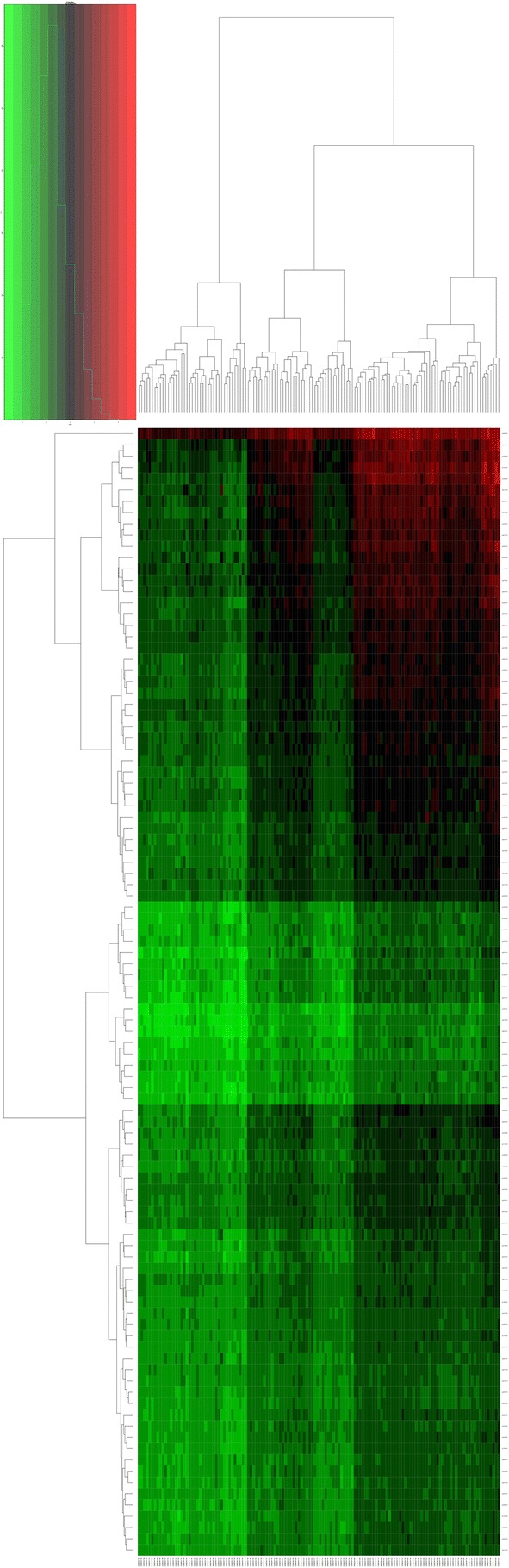
Heat map of the top 50 (DMPs) in GSE76938

Data from each microarray were separately analysed by R software to obtain the DEGs or DMGs. A total of 6208 DEGs were obtained from the microarray data, including 3503 upregulated genes and 2705 downregulated genes. We identified 2382 hypermethylated and 4120 hypomethylated genes in a comparative analysis of normal tissue and tumour samples in GSE76938. We then overlapped the aberrantly methylated genes and DEGs and identified a common list of 536 hypomethylated genes expressed at high levels and 322 hypermethylated genes expressed at low levels. To further explore aberrantly methylated DEGs, we overlapped the hypomethylated high expressed genes with oncogenes and identified 33 hypomethylated highly expressed oncogenes. These findings suggest that aberrant methylation contributes to the high expression of oncogenes in PCa and subsequently promotes prostate tumourigenesis. Forty-two hypermethylated TSGs with low expression were identified by overlapping hypermethylated genes with low expression and TSGs, suggesting that hypermethylation results in the inhibition of TSGs in PCa, thereby promoting prostate tumourigenesis (Fig. 1). A representative heat map of GSE29079 (with the top 50 DEGs) is shown in Fig. 2. The top 50 DMGs between PCa tissue and normal tissue are represented in the heat map shown in Fig. 3.

### GO functional enrichment analysis

GO enrichment analysis was conducted using the DAVID, and the results are illustrated in Table 1. For hypomethylated highly expressed genes, the terms enriched in the BP category included negative regulation of transcription from the RNA polymerase II promoter, osteoblast differentiation, intracellular signal transduction, the Wnt signalling pathway, and actin cytoskeleton organization. The GO cell component category revealed enrichment in the nucleoplasm, cytosol, nuclear body, cytoplasm, and Golgi cisterna membrane. In addition, the molecular function category showed enrichment for factors involved in transcriptional regulation of DNA binding, ligase activity, and GTPase activator and transcription coactivator activity. Hypermethylated genes with low expression showed enrichment in the BP category in processes such as positive regulation of MAPK cascade and phosphatidylinositol 3-kinase signalling, muscle contraction, ageing, and signal transduction. The enriched terms in the cell component category mainly included focal adhesion, cell surface, sarcolemma, and extracellular exosome and space. Additionally, the enriched molecular functions were focused on actin, protein, PDZ domain binding, glycosaminoglycan binding, and transmembrane receptor protein tyrosine kinase activity.

**Table 1:** Gene Ontology analysis of aberrantly methylated differentially expressed genes in prostate cancer

Category	Term	Count	*P* value
Hypomethylated with high expression			
GOTERM_BP_DIRECT	GO:0000122 —negative regulation of transcription from RNA polymerase II promoter	41	5.49E−05
GOTERM_BP_DIRECT	GO:0001649 —osteoblast differentiation	12	1.99E−04
GOTERM_BP_DIRECT	GO:0035556 —intracellular signal transduction	25	6.61E−04
GOTERM_BP_DIRECT	GO:0007223 —Wnt signalling pathway, calcium-modulating pathway	7	8.00E−04
GOTERM_BP_DIRECT	GO:0030036 —actin cytoskeleton organization	12	0.0013
GOTERM_CC_DIRECT	GO:0005654 —nucleoplasm	111	8.73E−05
GOTERM_CC_DIRECT	GO:0005829 —cytosol	126	1.97E−04
GOTERM_CC_DIRECT	GO:0016604 —nuclear body	6	0.0021
GOTERM_CC_DIRECT	GO:0005737 —cytoplasm	176	0.0030
GOTERM_CC_DIRECT	GO:0032580 —Golgi cisterna membrane	8	0.0054
GOTERM_MF_DIRECT	GO:0005515 —protein binding	297	2.57E−04
GOTERM_MF_DIRECT	GO:0044212 —transcription regulatory region DNA-binding activity	16	0.0016
GOTERM_MF_DIRECT	GO:0016874 —ligase activity	18	0.0027
GOTERM_MF_DIRECT	GO:0005096 —GTPase activator activity	18	0.0037
GOTERM_MF_DIRECT	GO:0003713 —transcription coactivator activity	16	0.0066
Hypermethylated with low expression			
GOTERM_BP_DIRECT	GO:0043410 —positive regulation of MAPK cascade	8	8.37E−05
GOTERM_BP_DIRECT	GO:0006936 —muscle contraction	9	1.41E−04
GOTERM_BP_DIRECT	GO:0007568 —ageing	11	2.19E−04
GOTERM_BP_DIRECT	GO:0007165 —signal transduction	37	2.44E−04
GOTERM_BP_DIRECT	GO:0014068 —positive regulation of phosphatidylinositol 3-kinase signalling	7	3.77E−04
GOTERM_CC_DIRECT	GO:0005925 —focal adhesion	24	1.01E−03
GOTERM_CC_DIRECT	GO:0009986 —cell surface	25	1.34E−03
GOTERM_CC_DIRECT	GO:0070062 —extracellular exosome	74	2.77E−03
GOTERM_CC_DIRECT	GO:0042383 —sarcolemma	9	3.60E−03
GOTERM_CC_DIRECT	GO:0005615 —extracellular space	41	1.24E−02
GOTERM_MF_DIRECT	GO:0003779 —actin binding	16	1.37E−04
GOTERM_MF_DIRECT	GO:0005515 —protein binding	184	7.99E−04
GOTERM_MF_DIRECT	GO:0030165 —PDZ domain binding	8	8.30E−04
GOTERM_MF_DIRECT	GO:0005539 —glycosaminoglycan binding	4	0.0037
GOTERM_MF_DIRECT	GO:0004714 —transmembrane receptor protein tyrosine kinase activity	5	0.0044

### KEGG pathway analysis

The results of the KEGG pathway enrichment analysis implied that hypomethylated highly expressed genes were significantly enriched in AMPK signalling, cancer, and adherens junction pathways. Hypermethylated genes expressed at low levels demonstrated enrichment in arrhythmogenic right ventricular cardiomyopathy (ARVC), focal adhesion, hypertrophic cardiomyopathy (HCM), dilated cardiomyopathy, and PI3K-Akt signalling pathways (Table 2).

**Table 2:** Kyoto Encyclopedia of Genes and Genomes pathway analysis of aberrantly methylated differentially expressed genes in prostate cancer

Category	Term	Count	*P* value	Gene
Hypomethylated with high expression				
KEGG_PATHWAY	hsa04152: AMPK signalling pathway	12	0.0025	*AKT1, IGF1R, PDPK1, IRS2, CCND1, TSC2, ACACA, FASN, FBP1, EEF2, PPP2R2D, RPTOR*
KEGG_PATHWAY	hsa05200: pathways in cancer	24	0.0062	*HSP90AB1, DVL3, CTBP1, ADCY1, COL4A1, ADCY7, RXRA, CREBBP, GNA12, LEF1, FZD4, TCF7L2, TPM3, DAPK1, AKT1, JUP, IGF1R, WNT7B, CBLB, CCND1, GNB2, GNB1, CASP8, GNG7*
KEGG_PATHWAY	hsa04520: adherens junction	8	0.0094	*ACTG1, IGF1R, TJP1, WASF3, BAIAP2, CREBBP, LEF1, TCF7L2*
Hypermethylated with low expression				
KEGG_PATHWAY	hsa05412: arrhythmogenic right ventricular cardiomyopathy (ARVC)	8	3.99E−04	*DES, SGCG, DMD, ITGA7, ITGA1, CACNB2, GJA1, CTNNB1*
KEGG_PATHWAY	hsa04510: focal adhesion	12	0.0020	*CAV2, ITGA7, ITGA1, PDGFC, PTEN, FLNA, PIK3R1, MYLK, COL4A6, KDR, CTNNB1, PRKCB*
KEGG_PATHWAY	hsa05410: hypertrophic cardiomyopathy (HCM)	7	0.0038	*DES, SGCG, DMD, ITGA7, ITGA1, CACNB2, TPM1 1*
KEGG_PATHWAY	hsa05414: dilated cardiomyopathy	7	0.0054	*DES, SGCG, DMD, ITGA7, ITGA1, CACNB2, TPM1*
KEGG_PATHWAY	hsa04151: PI3K-Akt signalling pathway	15	0.0063	*FGFR2, ITGA1, KITLG, KIT, LPAR1, PTEN, COL4A6, KDR, EIF4B, CDKN1B, ITGA7, ANGPT1, PDGFC, FGF2, PIK3R1*

### PPI network construction and cytoHubba analysis

**Fig. 4:**
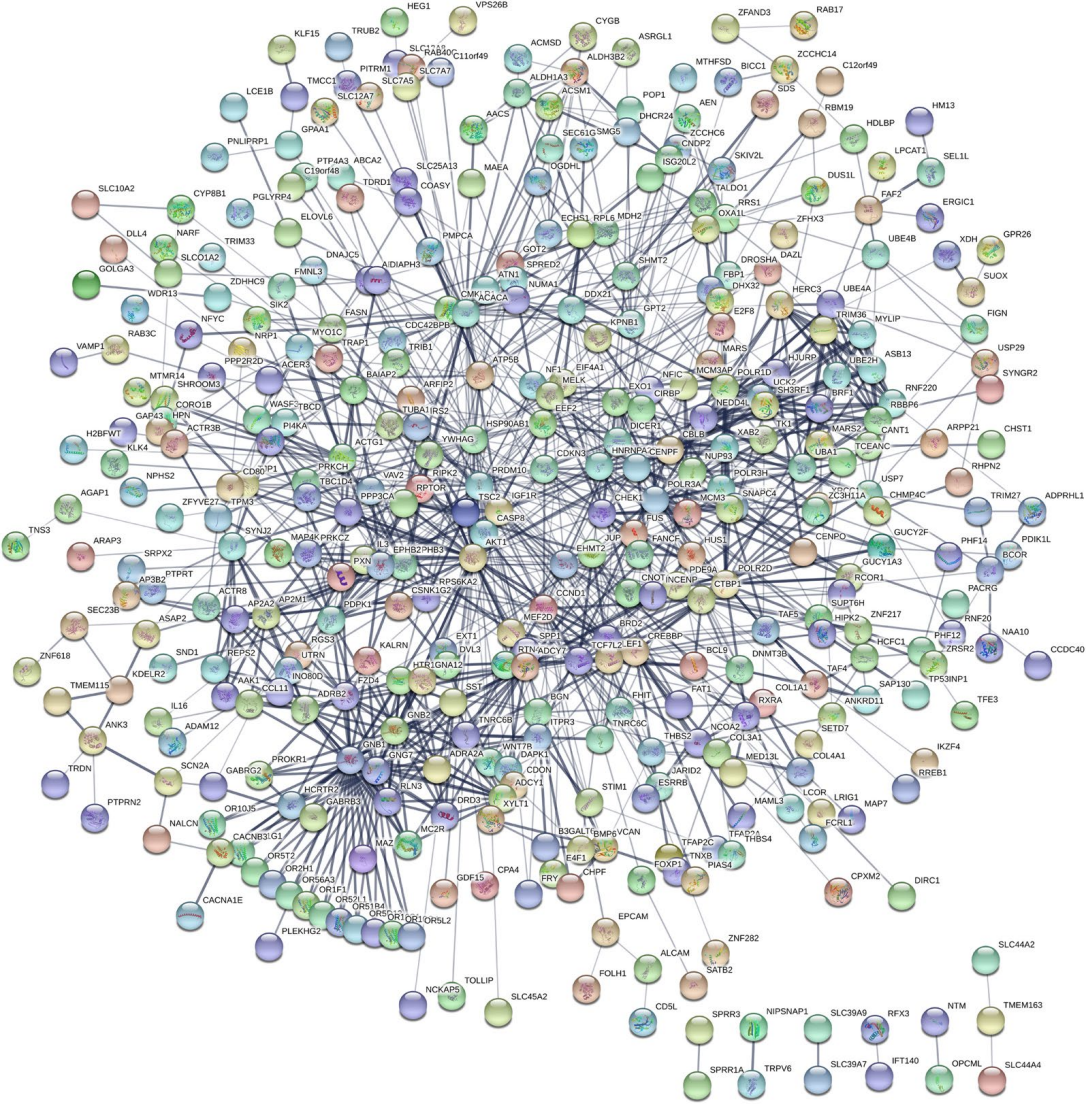
Protein–protein interaction (PPI) network of the upregulated hypomethylated genes

**Fig. 5:**
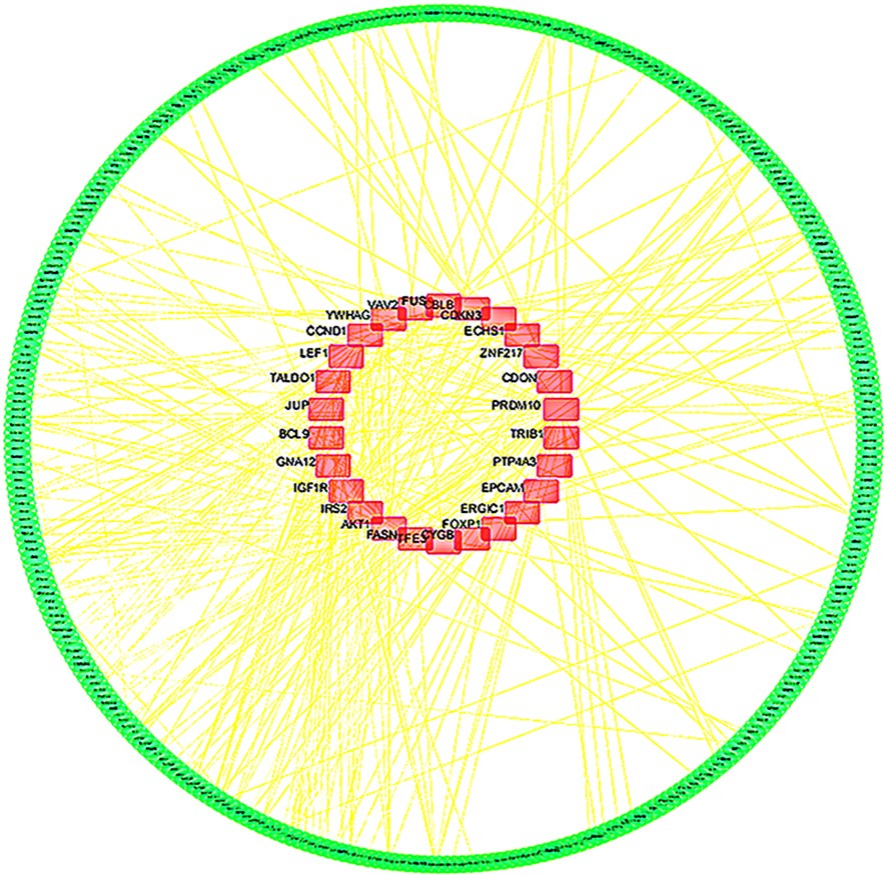
Protein–protein interaction (PPI) network of the upregulated hypomethylated oncogenes and their related genes

**Fig. 6:**
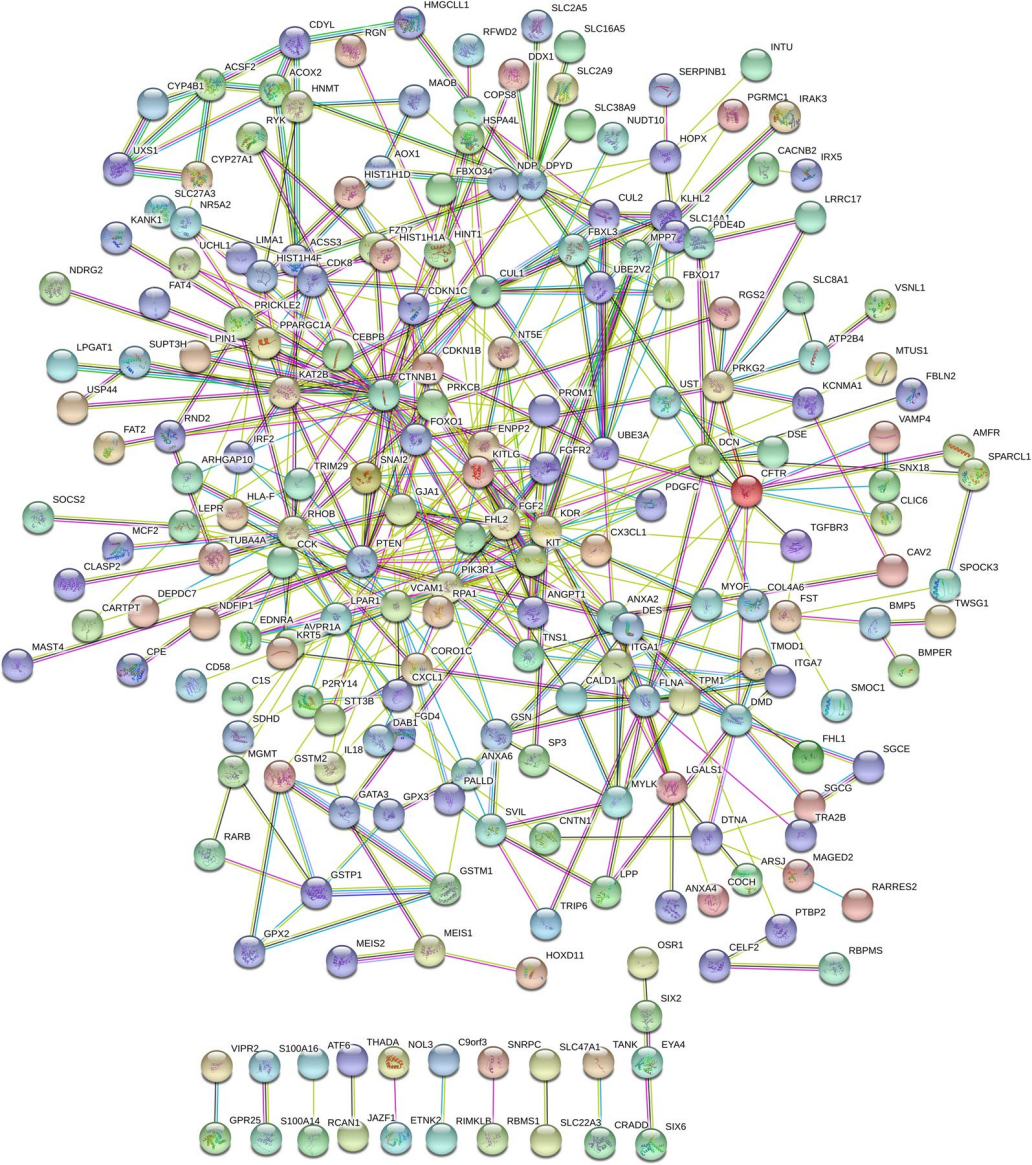
Protein–protein interaction (PPI) network of the downregulated hypermethylated genes

**Fig. 7:**
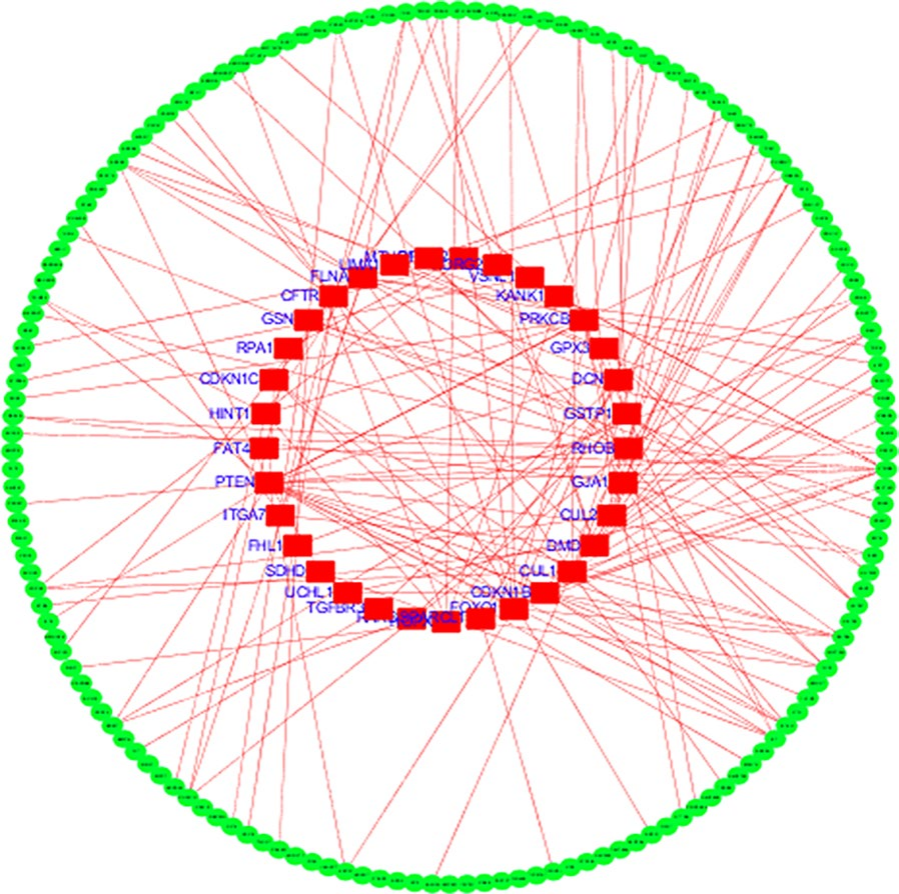
Protein–protein interaction (PPI) network of the upregulated hypomethylated TSGs and their related genes

The STRING was used to construct PPI networks. Ultimately, 264 nodes and 456 edges were established from the hypomethylated genes with high expression, and 159 nodes and 290 edges were established from the hypermethylated genes with low expression. For the upregulated hypomethylated oncogenes, the PPI network included 380 nodes and 1170 edges (Figs. 4 and 5). The PPI network of the downregulated hypermethylated TSGs is illustrated in Figs. 6 and 7 with 212 nodes and 458 edges. We then used cytoHubba to select hub genes. Upon overlapping the top 30 genes according to 5 ranking methods in cytoHubba, 2 downregulated hypermethylated genes (*FLNA* and *PRKCB*) and 4 upregulated hypomethylated genes (*AKT1*, *PRDM10*, *CCND1* and *FASN*) were chosen (Table 3).

**Table 3:** Hub genes among the aberrantly methylated differentially expressed genes ranked in cytoHubba

Category	Rank method in cytoHubba	Top 30 genes
Hypomethylated with high expression	Betweenness	***AKT1, PRDM10,*** *ACACA, GNB1, DICER1, CTBP1, ACTG1, CREBBP, HSP90AB1, DCY7, GNG7, PXN, POLR2D, PPP3CA, EEF2, BGN, GNB2, EHMT2,* ***FASN, CCND1*** *, OXA1L, SHMT2, CBLB, ATP5B, UBE4A, RPL6, SYNJ2, TCF7L2, EPHB2, FAF2*
	Bottleneck	***AKT1, PRDM10*** *, ACACA, DICER1, GNB1, ADCY7, CTBP1, SH3RF1, EHMT2, CREBBP, PPP3CA, ACTG1,* ***FASN*** *, RPL6, JUP, OXA1L, BGN, HSP90AB1, PXN, ECHS1, ATP5B, GPT2, POLR2D,* ***CCND1*** *, SYNJ2, SHMT2, EIF4A1, USP7, CHEK1, EEF2*
	Closeness	***AKT1, PRDM10,*** *ACACA, GNB1,* ***CCND1*** *, CTBP1, GNG7, DICER1, CREBBP, ADCY7, GNB2, PXN, IGF1R, PPP3CA, HSP90AB1, LEF1, EIF4A1, PDPK1, ITPR3, RPTOR, EHMT2, CHEK1, ACTG1, POLR2D, YWHAG, TCF7L2, EEF2,* ***FASN*** *, EPHB2, POLR3A*
	Radiality	***AKT1, PRDM10*** *, ACACA,* ***CCND1,*** *CTBP1, DICER1, CREBBP, PPP3CA, IGF1R, GNB1, ADCY7, LEF1, PXN, EIF4A1, GNB2, HSP90AB1, GNG7, RPTOR, ITPR3, YWHAG, PDPK1, CHEK1, EHMT2, ACTG1, CASP8, TJP1,* ***FASN*** *, TSC2, EPHB2, TCF7L2*
	Stress	***AKT1, PRDM10*** *, ACACA, GNB1, DICER1, CTBP1, ADCY7, GNG7, HSP90AB1, CREBBP, ACTG1, GNB2, POLR2D, PPP3CA, PXN, CBLB, EEF2,* ***CCND1*** *, EHMT2, BGN, TCF7L2, UBE4A, ATP5B, IGF1R, SYNJ2, POLR3A,* ***FASN*** *, SHMT2, EIF4A1, CHEK1*
Hypermethylated with low expression	Betweenness	*CTNNB1, DPYD, FGF2, VCAM1, PTEN, KIT, CFTR, RHOB, DCN, KDR, DMD, ANXA2,* ***PRKCB*** *, PRKG2, DES,* ***FLNA*** *, FST, KAT2B, PIK3R1, ITGA1, FOXO1, TPM1, PDE4D, ACSS3, CCK, KITLG, NT5E, CUL1, GATA3, HOPX*
	Closeness	*CTNNB1, FGF2, PTEN, KIT, VCAM1, KDR, PIK3R1, FOXO1, KITLG, DPYD, PROM1, CDKN1B, RHOB, LEPR, GJA1,* ***PRKCB*** *, KAT2B, ANXA2, DCN, SNAI2, DES, FGFR2, ITGA1, CUL1,* ***FLNA*** *, NT5E, CFTR, CCK, ANGPT1, PRKG2*
	Degree	*CTNNB1, FGF2, PTEN,KIT, VCAM1, KDR, KITLG, PIK3R1, KAT2B, CUL1, DPYD, RHOB, FOXO1,* ***FLNA*** *, CDKN1B, DES, DCN,* ***PRKCB,*** *ITGA1, DMD, CCK, CFTR, ANXA2, PROM1, UBE3A, TPM1, PRKG2, NT5E, LEPR, FGFR2*
	Eccentricity	*VCAM1, ANXA2, KIT, FGF2, KDR, PIK3R1, DMD, GJA1,* ***PRKCB****, PRKG2, TMOD1, ITGA1, COCH, CCK, AVPR1A, MTUS1, EDNRA, MYOF,* ***FLNA****, CFTR, CXCL1, LPAR1, ANXA6, GSN, PDE4D, NT5E, COL4A6, HLA*-*F, TRIM29, DAB1*
	Edge percolated component (EPC)	*FGF2, CTNNB1, KIT, PTEN, KDR, VCAM1, KITLG, PIK3R1, PROM1, CDKN1B, FOXO1, CUL1, KAT2B, ITGA1, FGFR2, ANGPT1, GJA1, DES, SNAI2, RHOB, UBE3A,* ***FLNA, PRKCB*** *, CCK, LEPR, CUL2, NT5E, TNS1, DCN, ANXA2*

The italics are hub oncogenes and TSGs

### Module analysis

**Fig. 8:**
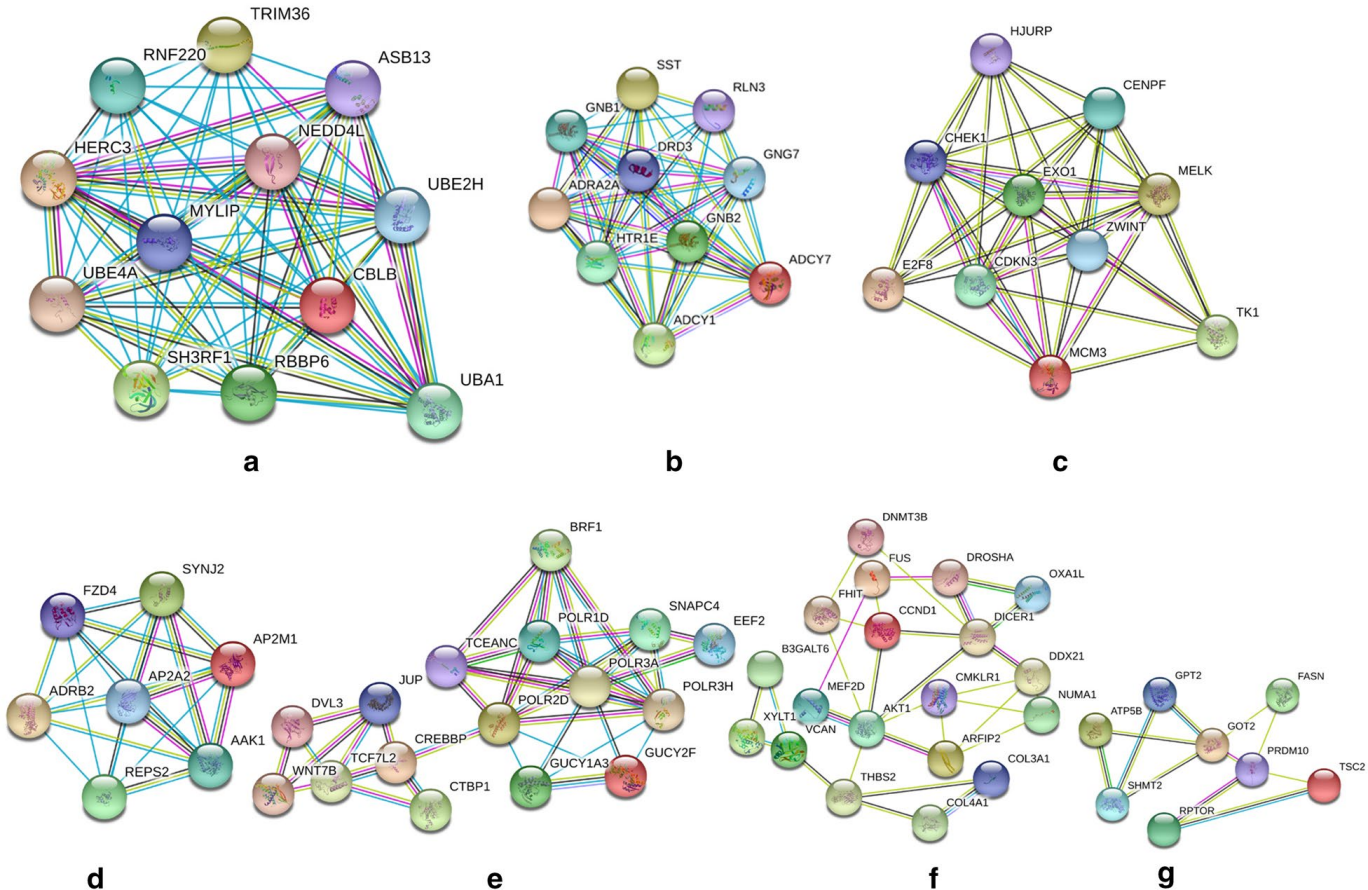
Seven upregulated hypomethylated gene modules. Module A (**a**) includes genes *TRIM36, HERC3, NEDD4L, UBE4A, CBLB, SH3RF1, ASB13, MYLIP, RNF220, UBA1, UBE2H, RBBP6*. For module B (**b**), there are ten genes in the module including *ADCY7, GNB1, GNB2, GNG7, ADCY1, DRD3, HTR1E, SST, RLN3, ADRA2A*. Module C (**c**) contains ten nodes with genes *CENPF, E2F8, TK1, HJURP, MCM3, ZWINT, MELK, CDKN3, EXO1, CHEK1*. Module D (**d**) has seven genes (*FZD4, REPS2, ADRB2, SYNJ2, AP2M1, AP2A2, AAK1*). Module E (**e**), F (**f**), and G (**g**) contains 16, 19, and 18 genes, respectively

**Fig. 9:**
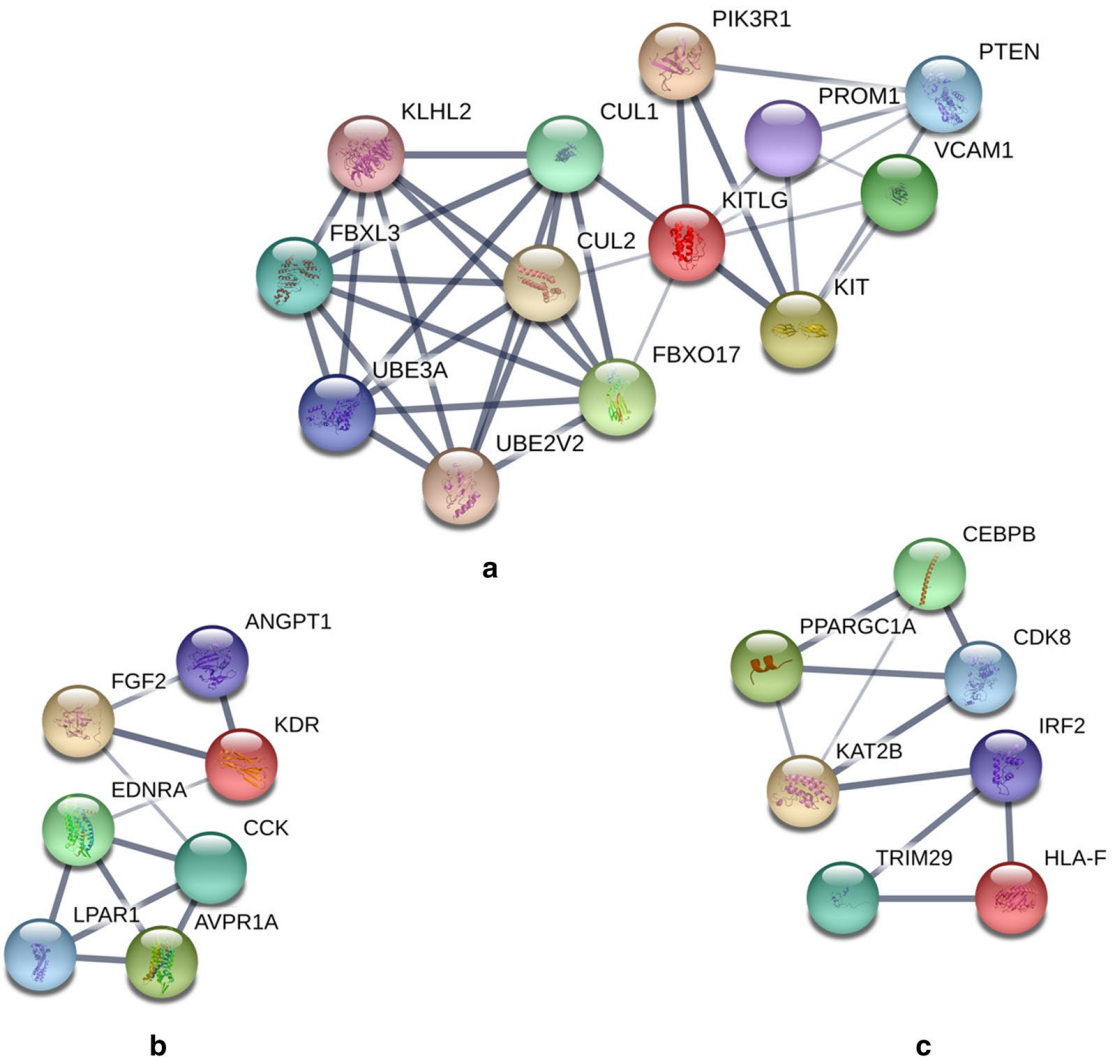
Three downregulated hypermethylated gene modules. Module A (**a**) included genes *CUL2, PIK3R1, UBE3A, PTEN, PROM1, FBXL3, KITLG, KIT, KLHL2, FBXO17, CUL1, VCAM1, UBE2V2*. For module B (**b**), there are seven genes in the module including *ANGPT1, FGF2, LPAR1, EDNRA, KDR, AVPR1A, CCK*. Module C (**c**) contains seven nodes with genes *HLA-F, IRF2, PPARGC1A, TRIM29, CDK8, CEBPB, KAT2B*

Overall, 7 modules in the network of hypomethylated genes with high expression and 3 modules in the network of hypermethylated genes with low expression were statistically significant (Figs. 8 and 9). The GO terms and KEGG pathways were then analysed (Table 4). The results of the pathway enrichment analysis implied that hypomethylated highly expressed genes were significantly enriched in pathways associated with ubiquitin-mediated proteolysis, GABAergic synapses, the cell cycle, endocytosis, purine metabolism, focal adhesion, and biosynthesis of amino acids. Hypermethylated genes with low expression demonstrated enrichment in cancer signalling and Rap1 signalling pathways.

**Table 4:** Module analysis of the protein–protein interaction network

Category	Module	Score	Nodes	Enrichment and pathway description	Genes
Hypomethylated with high expression	1	12.00	12	GO:0016874—ligase activity GO:0004842—ubiquitin protein transferase activity GO:0000209—protein polyubiquitination hsa04120: ubiquitin-mediated proteolysis GO:0071377—cellular response to glucagon stimulus	*TRIM36, HERC3, NEDD4L, UBE4A, CBLB, SH3RF1, ASB13, MYLIP, RNF220, UBA1, UBE2H, RBBP6*
	2	10.00	10	GO:0007186—G-protein-coupled receptor signalling pathway GO:0007193—adenylate cyclase-inhibiting G-protein-coupled receptor signalling pathway hsa04727: GABAergic synapse	*ADCY7, GNB1, GNB2, GNG7, ADCY1, DRD3, HTR1E, SST, RLN3, ADRA2A*
	3	8.889	10	GO:0005634—nucleus GO:0005515—protein binding GO:0006260—DNA replication hsa04110: cell cycle	*CENPF, E2F8, TK1, HJURP, MCM3, ZWINT*, *MELK, CDKN3, EXO1, CHEK1*
	4	7.00	7	GO:0030669—clathrin-coated endocytic vesicle membrane GO:0060071—Wnt signalling pathway, planar cell polarity pathway GO:0035615—clathrin adaptor activity hsa04144: endocytosis	*FZD4, REPS2, ADRB2, SYNJ2, AP2M1, AP2A2, AAK1*
	5	5.20	16	GO:0006383—transcription from RNA polymerase III promoter GO:0003899—DNA-directed RNA polymerase activity GO:0032481—positive regulation of type I interferon production hsa00230: purine metabolism	*POLR2D, GUCY2F, TCF7L2, POLR1D, EEF2, POLR3H, GUCY1A3, SNAPC4, TCEANC, JUP, WNT7B, CTBP1, DVL3, BRF1, CREBBP, POLR3A*
	6	3.444	19	GO:0001649—osteoblast differentiation GO:0030206—chondroitin sulfate GO:0005515—protein binding hsa05222: focal adhesion	*FHIT, COL4A1, DROSHA, NUMA1, DDX21, CMKLR1, COL3A1, DICER1, B3GALT6, OXA1L, MEF2D, FUS, DNMT3B, ARFIP2, VCAN, THBS2, CCND1, XYLT1, AKT1*
	7	3.143	8	GO:0009058—biosynthetic process GO:0005759—mitochondrial matrix GO:0030170—pyridoxal phosphate binding hsa01230: biosynthesis of amino acids	*GPT2, FASN, GOT2, RPTOR, ATP5B, TSC2, PRDM10, SHMT2*
Hypermethylated with low expression	1	6.00	13	GO:0016032—viral process GO:0048015—phosphatidylinositol-mediated signalling GO:0061630—ubiquitin protein ligase activity hsa05200: pathways in cancer	*CUL2, PIK3R1, UBE3A, PTEN, PROM1, FBXL3, KITLG, KIT, KLHL2, FBXO17, CUL1, VCAM1, UBE2V2*
	2	3.667	7	GO:0007202—activation of phospholipase C activity GO:0010595—positive regulation of endothelial cell migration GO:0008284—positive regulation of cell proliferation	*ANGPT1, FGF2, LPAR1, EDNRA, KDR, AVPR1A, CCK*
	3	3.333	7	hsa04015: Rap1 signalling pathway GO:0045944—positive regulation of transcription from RNA Polymerase II promoter GO:0005654—nucleoplasm	*HLA-F, IRF2, PPARGC1A, TRIM29, CDK8, CEBPB, KAT2B*

### Identification and validation of the six selected genes

We next used TCGA to validate our results. The outcome is summarized in Table 5. We found that methylation and expression statuses were also significantly altered in TGCA data, consistent with our findings. However, the expression of *CCND1* was downregulated in tumour samples compared to normal samples. This finding needs to be confirmed by further experiments. With regards to methylation status, *PRKCB* was hypomethylated.

**Table 5:** Validation of the hub genes in TCGA

Category	Hub gene	Expression status	*P* value	Methylation status	*P* value
Hypomethylated with high expression	*AKT1*	Upregulated	0.0063	Hypomethylated	7.17E−07
	*PRDM10*	Upregulated	0.0031	Hypomethylated	4.62E−10
	*CCND1*	–	–	Hypomethylated	5.98E−19
	*FASN*	Upregulated	3.85E−15	Hypomethylated	6.36E−21
Hypermethylated with low expression	*FLNA*	Downregulated	2.05E−29	Hypermethylated	6.99E−20
	*PRKCB*	Downregulated	3.54E−37	–	–

### Genetic alteration related to the hub genes

**Fig. 10:**
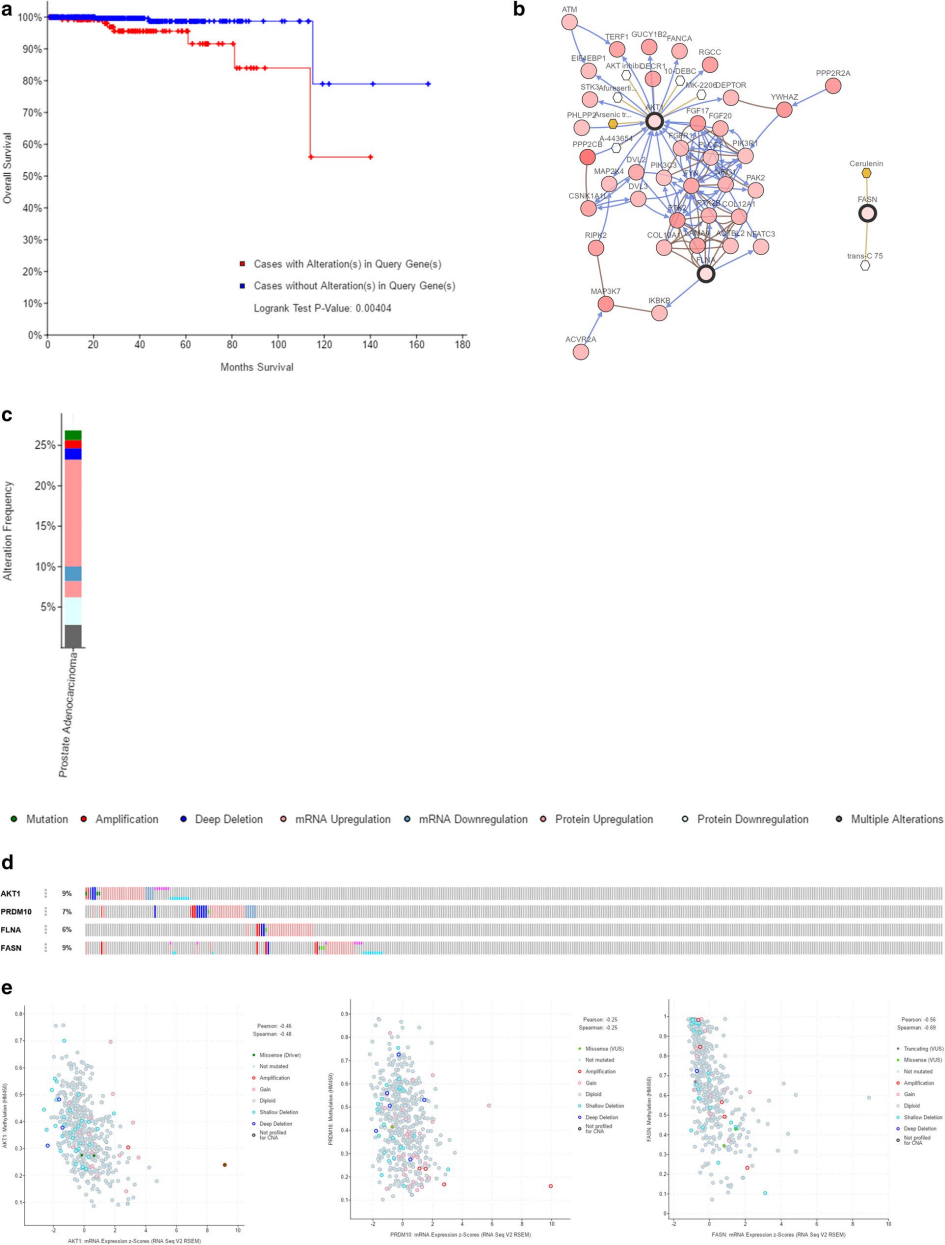
Genetic alterations connected to the hub genes. **a** indicates the relationship between hub genes and OS. **b** illustrates a network constructed with our 4 hub genes, their 50 most frequently neighbouring genes, and drugs targeting the hub genes. **c**, **d** shows the alteration of the hub genes. **e** points out the correlations between mRNA expression and DNA methylation for the hub genes in the TCGA dataset

**Fig. 11:**
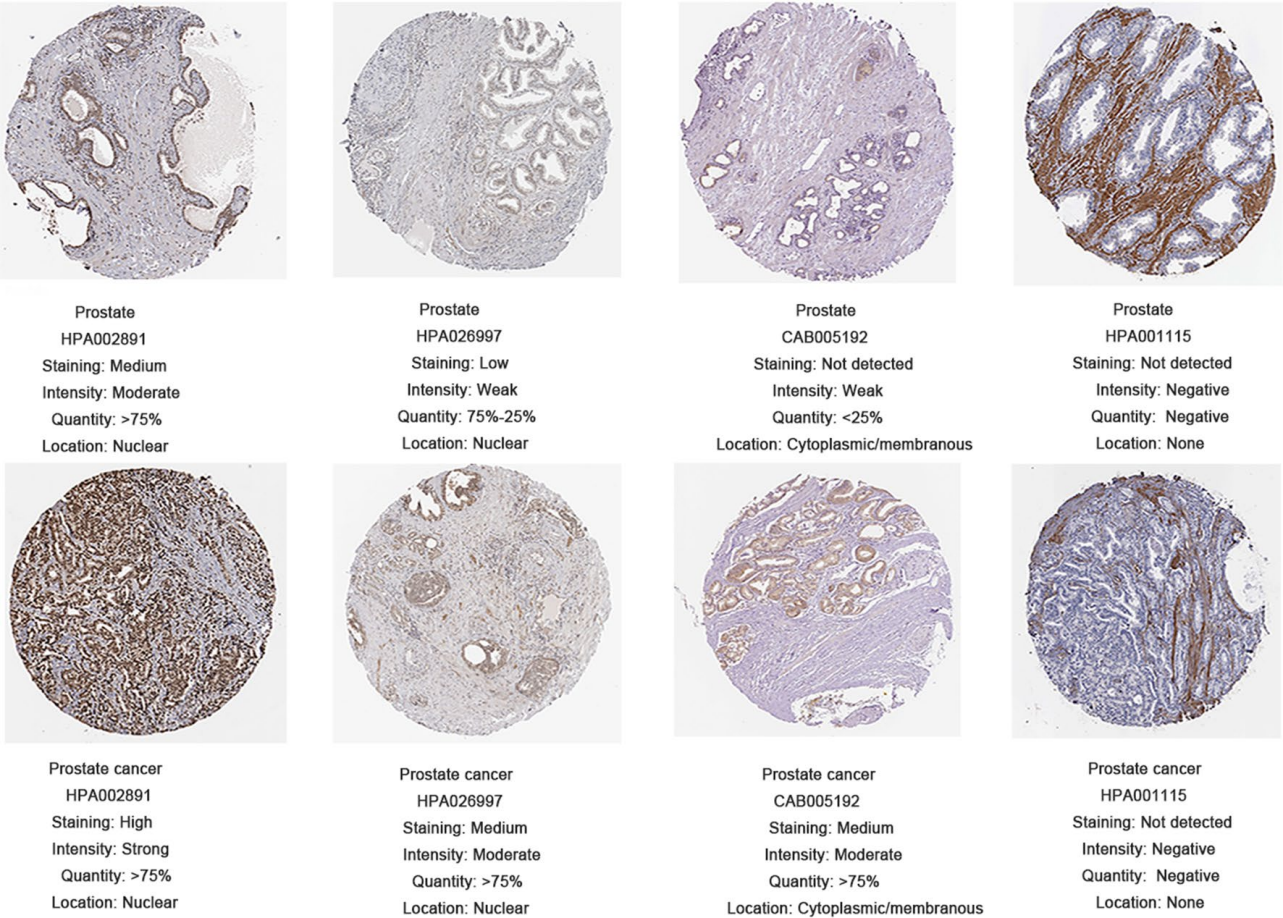
Validation of the hub genes using the Human Protein Atlas (HPA) database

**Fig. 12:**
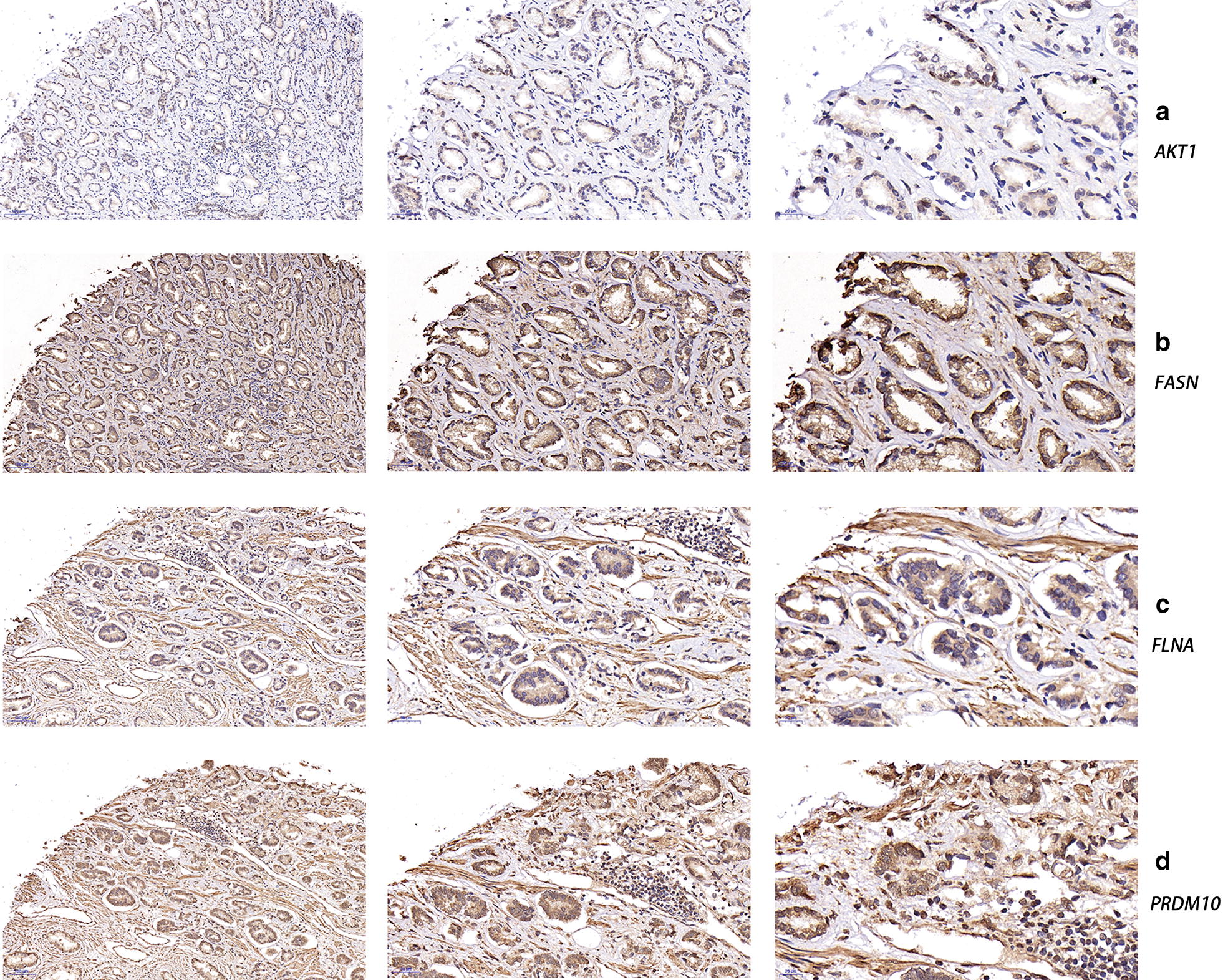
Representative microscopy images of prostate cancer (PCa) sections. **a**, **b**, **c**, **d** is the immunohistochemistry of *AKT1*, *FASN*,* FLNA* and *PRDM10*

We used cBioPortal software to explore genetic alteration related to the hub genes. We found that as a group, the hub genes were closely related to OS (Fig. 10a). We also observed a similar trend between hub genes and prognosis, although the relationship was not statistically significant. Figure 10b illustrates a network constructed with our 4 hub genes, their 50 most frequently altered neighbouring genes, and drugs targeting the hub genes (only 3 of the 4 had nodes or were targeted by drugs; the remaining gene, *PRDM10*, is not shown). Information on the alteration of the hub genes is exhibited in Fig. 10c, d. We found that these 4 hub genes were altered in 134 (27%) of the 498 sequenced cases/patients (499 total) and that *AKT1* and *FASN* most frequently exhibited alterations (9% each), including amplification and deep deletion. Figure 10e shows the correlations between mRNA expression and DNA methylation for the hub genes in the TCGA Prostate Adenocarcinoma (PRAD) patient dataset; the correlations were negative, indicating that methylation regulated the mRNA expression of these genes (except *FLNA*, for which there were insufficient data). This finding suggests that methylation plays an important role in the expression of these genes. The results of the validation of the hub genes on a translational level through the HPA database are shown in Fig. 11. Immunohistochemistry showed that *AKT1, FASN,* and *FLNA* protein was mainly expressed in the cytoplasm of PCa cells, while PRDM10 protein was mainly expressed in the nucleus (Fig. 12).

## Discussion

The initiation and development of PCa is a complex and multistage process regulated by genetic and epigenetic changes in pro-tumourigenic oncogenes and anti-tumourigenic TSGs. As with many other tumours, aberrant changes in epigenetic modifications, such as acetylation, phosphorylation, and in particular, DNA methylation, have been detected in PCa [16, 17]. Identifying novel biomarkers in PCa will contribute to improving the diagnosis, treatment, and prognostic assessment of PCa patients.

The GEO database is a free repository of microarray and next-generation sequencing analyses that was used to obtain expression profile (GSE29079) and methylation profile (GSE76938) datasets. R software can be used effectively for the analysis of genes in different groups of samples that are differentially expressed under various experimental conditions. In our study, 3 upregulated hypomethylated oncogenes and 1 downregulated hypermethylated TSG were identified. Functional enrichment analysis revealed that aberrant methylation affected certain pathways and hub genes. These results may offer novel insights into PCa pathogenesis.

DAVID analysis of upregulated hypomethylated genes demonstrated enrichment of the genes in biological processes such as regulation of RNA polymerase II-driven transcription, osteoblast differentiation, intracellular signal transduction, the Wnt signalling pathway, and actin cytoskeleton organization. This finding is consistent with previous studies that have shown that PCa cells stimulate the differentiation of pre-osteoplastic cells through regulators of bone metabolism, thereby facilitating prostate cancer metastasis to bones [18]. Additionally, the Wnt cascade can act as a master regulator by integrating signals from the PI3K/mTOR, MAPK, and AR pathways [19]. The MF category in GO analysis largely showed enrichments in protein and transcription regulatory region DNA binding, ligase activity, and GTPase activator and transcription coactivator activity. Previous studies have reported that GTPase activators regulate intercellular junctions and are disrupted during tumourigenesis [20]. KEGG pathway enrichment analysis suggested significant enrichment in AMPK signalling, cancer, and adherens junction pathways, consistent with the fact that the activated AMPK pathway is involved in the growth and survival of human PCa [21].

Downregulated hypermethylated genes in PCa were enriched for positive regulation of MAPK cascade and phosphatidylinositol 3-kinase signalling, muscle contraction, ageing, and signal transduction in the BP category. The MF category in GO analysis indicated involvement in actin binding, protein binding, PDZ domain binding, glycosaminoglycan binding and transmembrane receptor protein tyrosine kinase activity. Previous studies have shown that actin binding proteins can serve as key suppressors of cell migration and micrometastatic dissemination in PCa [22]. Additionally, tyrosine receptor kinase is an essential regulator of PCa proliferation and tumour growth [23]. KEGG pathway analysis revealed enrichment in ARVC, focal adhesion, HCM, dilated cardiomyopathy, and PI3K-AKT signalling pathways. The role of focal adhesion kinase (FAK) signalling in tumourigenesis and tumour progression has been extensively researched and has led to the development of FAK tyrosine kinase inhibitors as potential anticancer drugs [20]. Activation of the PI3K/AKT pathway has also been shown to play a major role in the aggressive nature of many prostate cancers [24]. Understanding the biological processes and signalling pathways in which aberrantly methylated DEGs are involved can help illuminate the pathogenesis of PCa and identify new therapeutic targets.

In the PPI network generated with Cytoscape, significantly more interactions than expected were observed for the aberrantly methylated DEGs. Importantly, a number of upregulated hypomethylated genes appeared to be involved in the tumourigenesis and tumour progression of PCa. We visualized the networks in Cytoscape, identified hub genes using cytoHubba, and validated the identified oncogenes *AKT1*, *PRDM10*, and *FASN* using the TCGA PRAD patient dataset. The serine/threonine kinase Akt, with 3 isoforms (Akt1, Akt2, and Akt3), plays a critical role in regulating diverse cellular functions, including cell growth, proliferation, survival, transcription, and protein synthesis [25, 26]. Akt1 expression is frequently elevated in breast and prostate cancers [27, 28]. Additionally, Akt1 appears to be robustly involved in the tumourigenesis and invasion of cancer cells [29]. Studies have shown that *Akt1* is almost completely hypomethylated in bladder cancer tissues [30]. However, the methylation pattern for *Akt1* in PCa tissue has not been defined. The rate of *Akt1* mutation in PCa was 9%; we hypothesize that the mutations cause the aberrant methylation and/or upregulation of *Akt1*. Akt1 is the target of the antitumour drug arsenic trioxide, which is currently used for patients with acute promyelocytic leukaemia [31]. Therefore, it is possible that Akt1 may serve as a potential drug target in other tumour types, including PCa.

*PRDM10* is a poorly studied member of the PRDM family. It lacks enzymatic activity and is believed to function as a transcriptional cofactor by recruiting histone-modifying enzymes to target promoters [32]. PRDM10 may serve as a transcriptional regulator for normal tissue differentiation and play important roles in promoting tumour development [33, 34]. In PCa, *PRDM10* has been found to be altered in approximately 7% of cases, indicating a similar mechanism may occur in PCa tumourigenesis. It has been reported that breast cancer cells show upregulated expression of PRDM10 associated with hypomethylation of the *PRDM10* gene, suggesting the involvement of this gene in the proliferation and invasion of breast cancer cells [35]. Similarly, we found that hypomethylation of *PRDM10* in PCa led to high expression in PCa samples, which may affect antitumour activity during tumour development. The *Fatty Acid Synthase (FASN)* protein-coding gene exhibits high expression levels in tumours, including PCa [36]. This gene has been shown to play critical roles in cancer progression and aggressiveness and to be highly associated with poor prognosis, high risk of disease recurrence, and drug resistance [37, 38]. In our study, we found that hypomethylation of *FASN* led to high expression of *FASN* in PCa, indicating that *FASN* may function as a promoter of PCa tumourigenesis. *FASN* was altered in approximately 9% of PCa patients; we hypothesize that the mutations may drive the high expression of *FASN* in PCa. In addition, the FASN-targeting drug cerulenin can dose-dependently decrease HER2/neu protein levels in breast cancer cells (from a 14% decrease at 1.25 mg/L to a 78% decrease at 10 mg/L), and it has been suggested as a possible anticancer treatment [39]. In this study, we identified 3 oncogenes that were highly expressed in PCa samples compared to normal tissues, which suggested that these genes may play vital roles in PCa occurrence. Aberrant methylation of these 3 oncogenes may lead to their upregulated expression in PCa.

We identified *FLNA* as a TSG that was hypermethylated and downregulated in PCa. It has been reported that FLNA is required for the regulation of cell migration and invasion [40]. However, emerging evidence suggests that it may also be involved in different tumourigenic processes, such as DNA damage and angiogenesis [41, 42]. Downregulation of this gene has been observed in a wide spectrum of human malignancies, including gastric cancers and renal cancers. FLNA has also been shown to be significantly correlated with lymph node metastasis, disease stage, histological grade, and poor OS through promotion of the degradation of MMP-9 [43, 44]. The methylation patterns of *FLNA* in PCa have not been previously described. In our study, we found that *FLNA* was hypermethylated and downregulated in PCa, suggesting that aberrant methylation of *FLNA* in PCa may lead to the deregulation of this TSG, thereby impacting tumour development.

Core module analysis of the PPI network for the upregulated hypomethylated genes suggested that ubiquitin-mediated proteolysis, GABAergic synapses, the cell cycle, endocytosis, purine metabolism, focal adhesion, and biosynthesis of amino acids may be involved in PCa progression. Studies have shown that ubiquitin-mediated proteolysis, endocytosis, and focal adhesion pathways can impact PCa development and progression in different ways [45–47]. In addition, in the GABAergic synapse pathway, GABA induces GRP secretion via GABBR1 in neuroendocrine-like cells, which is involved in PCa progression [48]. The cell cycle is a vital cellular process involving DNA replication and translation, and it tends to be deregulated in cancer [49]. However, the roles of purine metabolism and biosynthesis of amino acids in PCa, as well as the impact of aberrant methylation is unknown. In our study, the identified hypermethylated downregulated genes were enriched in cancer signalling and Rap1 signalling pathways. Cancer signalling pathways are fundamental for cancer development and sustained carcinogenesis. In addition, aberrant Rap1 activation leads to tumour progression, and it may be induced by cytokines such as galanin [50]. The specific manner in which aberrant methylation affects the functional roles of these pathways in PCa development and progression needs to be investigated in the future.

There were several limitations of the present study. First, the study focused on upregulated hypomethylated and downregulated hypermethylated genes. However, contra-regulated genes were not included; these need to be considered in the future. Second, validation of the aberrantly methylated genes was carried out with TCGA data using in silico approaches. Biological experiments will be necessary to validate these findings in the future. Third, our study was limited to only 2 datasets. Therefore, larger sample sizes are needed to validate the findings of this study. Lastly, more experiments, such as qRT-PCR experiments comparing expression in PCa tissues and normal tissues, should be conducted to confirm the target genes. We have collected PCa tissues and normal tissues, and the results of further analyses will be presented in the future.

## Conclusion

In summary, our results identified a series of aberrantly methylated differentially expressed oncogenes and TSGs and their associated pathways in PCa using integrated bioinformatic analysis of gene expression and gene methylation microarray datasets. These results may contribute to a more comprehensive understanding of the molecular mechanisms underlying the occurrence and development of PCa. The 4 hub genes found, namely, *AKT1*, *PRDM10*, *FASN*, and *FLNA,* were validated using the TCGA PRAD patient dataset. These genes, when aberrantly methylated, may serve as putative biomarkers for the precise diagnosis and treatment of PCa in the future. In comparison to other studies that have focused on individual datasets, our study analysed multiple datasets to produce more robust results regarding gene expression changes and gene modifications that are important in the development and progression of PCa. Future studies will be aimed at validating the functional significance of the identified hub genes in PCa.

## Abbreviations

PCa: prostate cancer
DEG: differentially expressed gene
DMG: differentially methylated gene
GO: gene ontology
KEGG: Kyoto Encyclopedia of Genes and Genomes
PPI: protein–protein interaction
STRING: Search Tool for the Retrieval of Interacting Genes
TCGA: the Cancer Genome Atlas
HPA: Human Protein Atlas
